# Effects of Stimulation of Soluble Guanylate Cyclase on Diabetic Nephropathy in Diabetic eNOS Knockout Mice on Top of Angiotensin II Receptor Blockade

**DOI:** 10.1371/journal.pone.0042623

**Published:** 2012-08-10

**Authors:** Ina M. Ott, Markus L. Alter, Karoline von Websky, Axel Kretschmer, Oleg Tsuprykov, Yuliya Sharkovska, Katharina Krause-Relle, Jens Raila, Andrea Henze, Johannes-Peter Stasch, Berthold Hocher

**Affiliations:** 1 Institute for Nutritional Science, University of Potsdam, Potsdam, Germany; 2 Center for Cardiovascular Research, Charité Campus Mitte, Berlin, Germany; 3 Department of Nephrology, Charité Campus Benjamin Franklin, Berlin, Germany; 4 Bayer HealthCare AG, Wuppertal, Germany; 5 Institute of Pharmacy, University of Halle-Wittenberg, Halle (Saale), Germany; Maastricht University, The Netherlands

## Abstract

The prevalence of diabetes mellitus and its complications, such as diabetic nephropathy (DN), is rising worldwide and prevention and treatment are therefore becoming increasingly important. Therapy of DN is particularly important for patients who do not adequately respond to angiotensin receptor blocker (ARB) treatment. Novel approaches include the stimulation of soluble guanylate cyclase (sGC) as it is reported to have beneficial effects on cardiac and renal damage. We aimed to investigate the effects of the sGC stimulator riociguat and ARB telmisartan on kidney function and structure in a hypertensive model of diabetic nephropathy.

Seventy-six diabetic male eNOS knockout C57BL/6J mice were randomly divided after having received streptozotocin: telmisartan (1 mg/kg/d), riociguat (3 mg/kg/d), riociguat+telmisartan (3+1 mg/kg/d), and vehicle. Fourteen mice were used as non-diabetic controls. Treatment duration was 11 weeks.

Glucose concentrations were increased and similar in all diabetic groups. Telmisartan insignificantly reduced blood pressure by 5.9 mmHg compared with diabetic controls (111.2±2.3 mmHg vs. 117.1±2.2 mmHg; p = 0.071). Treatment with riociguat both alone and in combination with telmisartan led to a significant reduction of blood pressure towards diabetic vehicle (105.2±2.5 mmHg and 105.0±3.2 mmHg, respectively, vs. 117.1±2.2 mmHg). Combined treatment also significantly decreased albuminuria compared with diabetic controls (47.3±9.6 µg/24 h vs. 170.8±34.2 µg/24 h; p = 0.002) reaching levels similar to those of non-diabetic controls (34.4±10.6 µg/24 h), whereas the reduction by single treatment with either telmisartan (97.8±26.4 µg/24 h) or riociguat (97.1±15.7 µg/24 h) was not statistically significant. The combination treatment led to a significant (p<0.01) decrease of tissue immunoreactivity of malondialdehyde, as consequence of reduced oxidative stress.

In conclusion, stimulation of sGC significantly reduced urinary albumin excretion in diabetic eNOS knockout mice treated already with ARB. Thus, this new drug class on top of standard ARBs administration may offer a new therapeutic approach for patients resistant to ARB treatment.

## Introduction

Worldwide prevalence of diabetes mellitus features increasing numbers hence medical consequences such as diabetic retinopathy, neuropathy and nephropathy extend as well. Taking into account that the latter is the leading cause of dialysis and kidney transplantation, prevention and treatment are becoming deliberately important [1]–[3]. Prevention of progressive diabetic nephropathy (DN) includes control of hyperglycaemia and blood pressure as well as additional nephroprotection such as inhibition of the renin-angiotensin-aldosterone system (RAAS) [4]–[7]. Telmisartan, which is an angiotensin receptor II blocker (ARB), has proven to be very effective at reducing transition rates to overt nephropathy [8] and increasing nitric oxide (NO) activity of the renal endothelium in patients with diabetes mellitus type 2 (T2DM) [6], [9]. Nevertheless there are a number of patients who do not adequately respond to this gold standard therapy with respect to blood pressure reduction and reduction of urinary albumin excretion, and the need for treatment improvement is highest in those patients. An animal model that resembles this clinical situation is the diabetic endothelial NO synthase (eNOS) knockout mouse [10]–[12].

DN is considered a progressive disease that is related to increased cardiovascular morbidity and mortality. There is evidence that endothelial dysfunction is associated with diabetic nephropathy [13], [14] and enhancement of NO bioavailability might improve the clinical outcome of these patients. Organic nitrates mimic the action of endogenous NO, but are unfeasible agents for chronic treatment due to development of tolerance. Consequently, new strategies for improved treatment are necessary [9].

The soluble guanylate cyclase (sGC), a key enzyme of the nitric oxide (NO) signaling pathway, is attracting rapidly growing interest as a therapeutic target in cardiovascular and pulmonary disease. On binding of NO to a prosthetic heme group on sGC, the enzyme catalyzes the synthesis of the second messenger cGMP, which produces vasorelaxation and inhibits smooth muscle proliferation, leukocyte recruitment, and platelet aggregation through a number of downstream mechanisms, including the activation of protein kinase, phoshodiesterases and ion-channels [15].

Riociguat (BAY 63-2521) is the first of a new class of drugs, i.e. the soluble guanylate cyclase stimulators which is currently in clinical development for several forms of pulmonary hypertension and heart disease. Riociguat has a dual mode of action: it sensitizes sGC to the body's own NO and can also increase sGC activity in the absence of NO, causing the aforementioned cGMP mediated effects [9]. In other words, riociguat significantly increases the activity of sGC independently of NO and has an even greater effect in synergy with NO [16]. This is thought to be important because impaired NO bioavailability is associated with reno-cardiovascular morbidity. In animal models of hypertension and of chronic renal failure, riociguat provided significant protection against cardiac and renal damage, respectively, reducing blood pressure, glomerulosclerosis, interstitial fibrosis, and left ventricular weight [17]. In a rodent model of chronic pressure and volume overload riociguat demonstrated anti-fibrotic tissue remodelling abilities [18]. With its novel mode of action, riociguat improves cardiac, pulmonary and renal hemodynamics and has the potential to overcome the limitations of currently approved therapies [9]. Nevertheless, effects of riociguat on progression of DN are still barely examined.

In order to test the potential effects of riociguat on the progression of DN, we chose diabetic eNOS knockout mice for our investigations since they well resemble the clinical situation in diabetic patients with nephropathy [10]–[12]. We have chosen our study design in a way that offers translation to clinical science of our data. Nowadays guideline based therapy of diabetic nephropathy includes treatment with ACE inhibitors or angiotension II receptor blockers (ARB). We decided thus to try to demonstrate efficacy on top of guideline based therapy in this model which is most close to the human disease in terms of pathological findings and clinical cause: Mice were treated for eleven weeks with the ARB telmisartan, riociguat, and both, respectively, and renal parameters, blood pressure and histology were assessed.

## Materials and Methods

This study was carried out in strict accordance with the recommendations in the “Guide for the Care and Use of Laboratory Animals of the National Institutes of Health”. The protocol was approved by the local animal welfare committee of the German State of Brandenburg (Landesamt für Verbraucherschutz, Landwirtschaft und Flurneuordnung, Frankfurt/Oder, Permit No. 23-2347-8-26-2008). All surgery was performed under inhalation anesthesia with isoflurane, and all efforts were made to minimize suffering.

### Chemicals

Riociguat [BAY 63-2521; Methyl 2-[1-2(-fluorobenzyl)-1*H*-pyrazolo[3,4-*b*]pyridin-3-yl]-4,6—diaminopyrimidin-5-ylmethylcarbamat] was synthesized by Bayer Pharma AG as described [19] and dissolved in Transcutol®/Cremophor®/water (10/20/70). Telmisartan and synthesized by Boehringer-Ingelheim Pharma GmbH & Co. KG (Ingelheim, Germany). Unless otherwise stated, all other reagents were of analytical grade and were purchased from Sigma-Aldrich (Seelze, Germany), Merck (Darmstadt, Germany) and Roth (Karlsruhe, Germany).

### Animal model and experimental design

Six-week old male eNOS (NOS3) knock out C57BL/6J mice (C57BL/6J-Nos3tm1Unc; n = 76) were obtained from The Jackson Laboratory (Bar Harbor, ME, USA). In accordance with local institutional guidelines for the care and use of laboratory animals, mice were housed under standardized conditions (12 h light/dark cycle, temperature of 23°C, humidity of 50–60%) and kept in solitary cages with commercial standard diet (Ssniff Spezialdiäten GmbH, Soest) and water *ad libitum*. Before induction of diabetes by streptozotocin (STZ), baseline measurement of blood pressure using non-invasive tail-cuff-method and metabolic cages were performed. Then mice intraperitoneally received STZ (100 mg/kg body weight) on 2 consecutive days and they were randomly divided into 4 treatment groups 1.5 weeks afterwards: telmisartan (1 mg/kg/d; n = 17); riociguat (3 mg/kg/d; n = 15); riociguat+telmisartan (3 mg/kg/d+1 mg/kg/d; n = 13); and vehicle (n = 17). Another 14 mice received vehicle after they had been administered citrate buffer instead of STZ in equal volume (non-diabetic vehicle controls). All substances were given once daily by oral gavage with equal volumes per body weight (daily record) for a period of 11 weeks. At week 5 and 8 blood glucose levels were measured to confirm hyperglycemia (>250 mg/dl). Final measurements in week 12 included experiments with metabolic cages, blood pressure recording and collecting urine and blood samples. At week 13 animals were sacrificed, organs weighted and harvested for histology. Urinary albumin was measured as described elsewhere [18] and urinary creatinine was measured by a Beckman DU 530 UV-VIS spectrophotometer (Beckman Coulter, Inc., Brea, Ca, USA) based on the Jaffé reaction of creatinine with alkaline picrate (creatinine detection kit, Dr. Lange Test, Dr. Bruno Lange GmbH, Berlin, Germany) according to the manufacturer's instructions. Furthermore plasma biomarkers cystatin C (Rules Based Medicine platform, Austin TX, USA), MCP-1, and TNF-α (Millipore platform, Oxfordshire, UK) were determined.

### Histological studies

Half of the left kidney of each mouse was fixed in 10% neutral buffered formalin, embedded in paraffin and cut into 3 µm sections. Sections were stained with Sirius Red, Periodic Acid-Schiff (PAS) and Elastica van Gieson. All microscopic examinations were performed in a blinded manner. Renal morphology (interstitial fibrosis, perivascular fibrosis, glomerulosclerosis and media–lumen ratio of blood vessels) was measured as recently described [20], [21]. In brief, glomerulosclerosis was defined by PAS-positive areas within the glomerulus, perivascular fibrosis by Sirius Red-positive material around arterial blood vessels using a subjective, semi-quantitative score system by two independent investigators. The severity of interstitial fibrosis was evaluated on the basis of Sirius red-stained material using computer-aided devices. 30 microscopic pictures per kidney section were transferred to a PowerMAC via Hitachi CCD camera and analysed by using ImageJ, an image-processing software (shareware from the NIH). Accordingly media-lumen ratio of blood vessels was determined by Elastica van Gieson staining. Area contents of the media and the lumen of intrarenal/intracardial arteries using ImageJ (shareware from the NIH) was determined. Afterwards, media/lumen ratio was calculated to serve as marker for arterial wall thickening.

### Immunohistochemistry

Deparaffinized kidney sections were quenched (0.3% H_2_O_2_ in methanol), blocked (1,5% serum in 1×PBS), and incubated with primary antibody as follows: rabbit anti-mouse TGFβ-1 (1∶50 dilution in 1.5% serum), Acris Antibodies GmbH, Herford, Germany), rabbit anti-mouse PAI-1 (1∶100, Santa Cruz Biotechnology, Inc., Santa Cruz, USA), and rat anti-mouse CD68 (1∶50, AbD Serotec, Oxford, UK). Overnight incubation at 4°C was followed by sequentially application of biotinylated goat anti-rabbit IgG (1∶200 dilution for TGFβ-1 and PAI-1; 1∶50 dilution for CD68), avidin and biotinylated horseradish peroxidase, and DAB chromogen using an ABC-staining system (Santa Cruz Biotechnology, Inc., Santa Cruz, USA). MDA-staining of deparaffinized kidney sections was performed using a goat anti-mouse antibody (1∶200 dilution in 1.5% serum, Immundiagnostik AG, Bensheim, Germany) and HRP-DAB System (R&D Systems Europe Ltd., Abingdon, UK) following the manufacturer's procedure.

Negative controls for immunostaining included omission of the corresponding primary antibody. All sections were lightly counterstained with hematoxylin.

Immunostaining was graded on blinded slides by two independent investigators. Glomerular expression of TGFβ-1 was carried out by using a semiquantitative score in 40–50 glomerular cross-sections taking into account absent, podocytic, and mesangial staining. Staining of CD68 was evaluated by counting positively stained macrophages in 30 glomeruli or by counting CD68-positive macrophages in 20 defined microscopic fields (0.04 mm^2^) omitting any glomeruli and vessels. Analysis of MDA stained kidney slices was done by analogy of interstitial fibrosis evaluation using 15 pictures of each animal.

### Statistical analyzes

All values are given as means ± standard error of the mean (SEM). Statistical analyzes were performed with SPSS 18.0 for Windows (SPSS Inc., Chicago, IL, USA). For comparisons between two groups of interest, the unpaired student's t test was used if variables were parametric and normally distributed, which was tested by Kolmogorow-Smirnov test. Otherwise, Wilcoxon-Mann-Whitney u test was used instead. Mortality calculations were performed using Kaplan-Meier analysis and Mantel-Cox log rank test. Differences were considered significant if p<0.05; and highly significant if p<0.01.

## Results

### Mortality

No significant differences among the study groups concerning mortality could be observed (p = 0.35 [log rank test]; data not shown).

### Blood pressure and blood glucose

Baseline blood pressure did not differ among the study groups in advance to STZ administration (Table 1). In contrast final blood pressure measurements revealed that riociguat alone and in combination with telmisartan significantly reduced blood pressure in comparison to diabetic control mice (p = 0.002 and p = 0.004, respectively), whereas administration of telmisartan alone led to a fairly modest and non-significant reduction of blood pressure (p = 0.071). Comparison between solely telmisartan treated and combined (riociguat and telmisartan) treated mice showed no significant differences in blood pressure. Blood glucose concentrations at week 5 and week 8 were similar in all diabetic groups independently of treatment and were highly increased compared with non-diabetic controls (data not shown).

**Table 1:** Experimental progress of blood pressure and blood glucose in diabetic eNOS knockout mice treated with riociguat (3 mg/kg/d), telmisartan (1 mg/kg/d), both (3 mg/kg/d and 1 mg/kg/d) or vehicle.

	Telmisartan	Riociguat	Riociguat + Telmisartan	Diabetic, vehicle	Control, vehicle
**Systolic blood pressure [mmHg]**					
Baseline	116.4±2.4	114.1±2.4	117.7±2.4	118.6±2.0	122.3±2.3
	n = 17	n = 15	n = 13	n = 17	n = 14
Final	111.2±2.3	105.2±2.5*	105.0±3.2*	117.1±2.2	118.8±5.1
	n = 14	n = 14	n = 12	n = 14	n = 14

Values are given as means ± SEM. For comparisons, student's t test was used.

* p<0.05 vs. diabetic vehicle.

Additionally non-diabetic eNOS knockout mice treated with vehicle were examined.

### Kidney function

**Figure 1:**
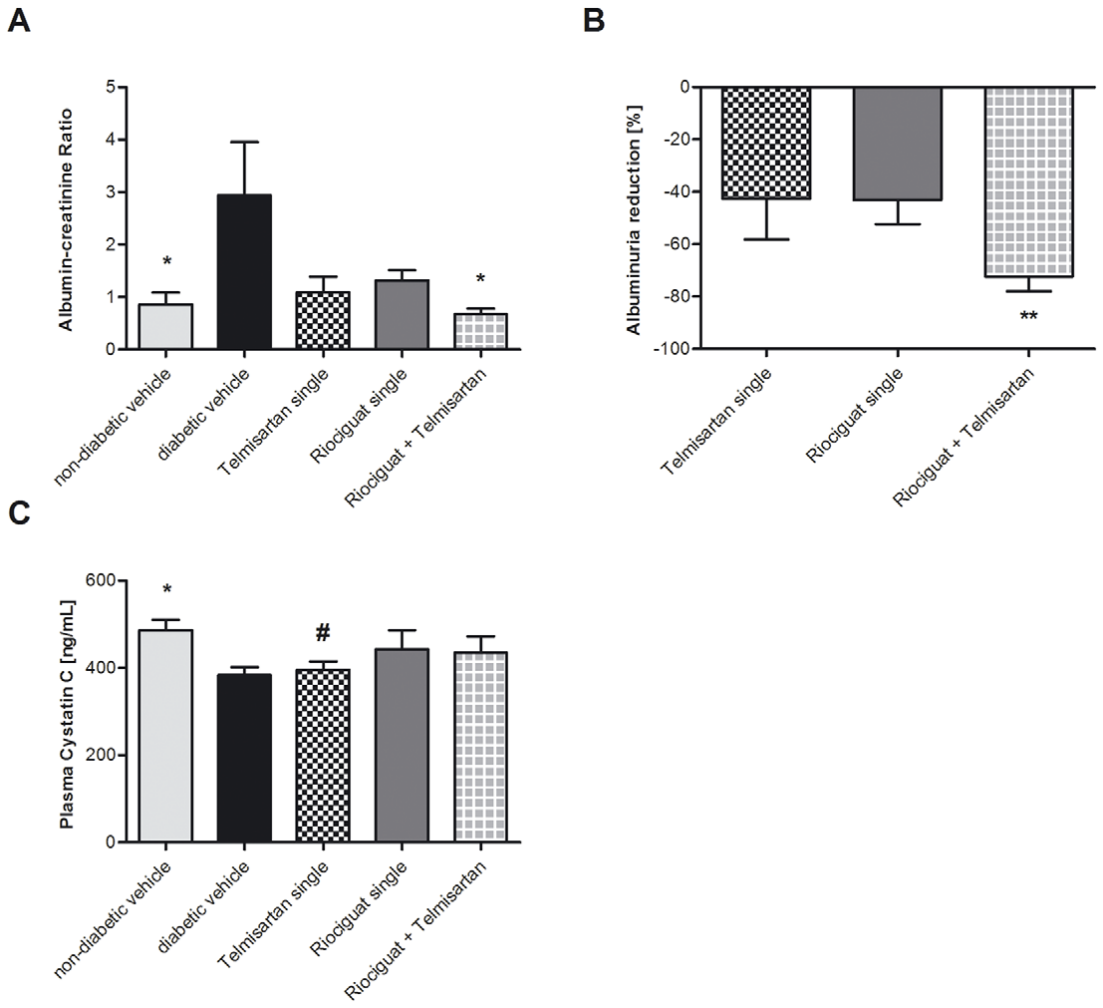
Significant reduction of albuminuria by combination therapy: Final kidney parameters in diabetic eNOS knockout mice treated with riociguat (3 mg/kg/d), telmisartan (1 mg/kg/d), both (3 mg/kg/d and 1 mg/kg/d) or vehicle, and non-diabetic eNOS knockout mice treated with vehicle, respectively. Urinary albumin-creatinine ratio (A), percentage reduction in urinary albumin excretion per day compared with diabetic controls (B) and cystatin C in Plasma (C). Values are given as means ± SEM. For comparisons, student's t test and Mann-Whitney u test, respectively, were used. ^*^ p<0.05 vs. diabetic vehicle; ^#^ p<0.05 vs. non-diabetic vehicle.

Therapy of diabetes with either riociguat or telmisartan alone did not lower urinary albumin excretion (p = 0.067 and p = 0.101, respectively; Table 2) nor albumine-creatinine ratio (p = 0.171 and p = 0.091, respectively; Figure 1A). However, the combined administration led to a significant reduction of urinary albumin excretion compared with diabetic control mice (47.3±9.6 µg/d vs. 31.4±10.1 µg/d; p = 0.003; Figure 1B), reaching levels similar to those of non-diabetic status. Furthermore, the combined treatment tended to reduce albuminuria as compared with animals treated solely with telmisartan, but failed significance, though (p = 0.090).

**Table 2:** Final urinary albumin as well as organ weights and histology of diabetic mice after 11 weeks of treatment with riociguat (3 mg/kg/d), telmisartan (1 mg/kg/d), riociguat and telmisartan (3 mg/kg/d and 1 mg/kg/d) or vehicle and of non-diabetic control mice, respectively.

	Telmisartan	Riociguat	Riociguat + Telmisartan	Diabetic, vehicle	Control, vehicle
**Parameter**					
Urinary Albumin [µg/24 h]	97.8±26.4	97.1±15.7	47.3±9.6*	170.8±34.2	31.4±10.6*
	n = 14	n = 13	n = 12	n = 13	n = 12
**Relative organ weights**					
Kidney [mg/g]	16.1±0.8	16.2±0.6	16.2±0.7	15.0±0.9	10.2±0.3*
	n = 14	n = 13	n = 12	n = 14	n = 14
Liver [mg/g]	60.4±1.3	62.2±1.4	61.1±1.3	60.4±1.1	42.7±1.0*
	n = 14	n = 13	n = 12	n = 14	n = 14
Heart [mg/g]	4.7±0.1	5.0±0.1	4.9±0.2	4.7±0.2	4.7±0.1
	n = 14	n = 13	n = 12	n = 14	n = 14
**Kidney histology**					
Interstitial fibrosis [%]	0.7±0.1	0.5±0.0*	0.5±0.1*	0.8±0.1	0.5±0.1*
	n = 14	n = 13	n = 12	n = 14	n = 14
Glomerulosclerosis [Score]	2.2±0.1	2.2±0.1	2.2±0.1	2.4±0.1	2.4±0.1
	n = 14	n = 13	n = 12	n = 14	n = 14
Nodular glomerulosclerosis [%]	4.4±0.8	4.4±0.7	5.0±0.8	7.1±1.3	2.3±0.6*
	n = 14	n = 13	n = 12	n = 14	n = 14

Values are given as means ± SEM. For comparisons, student's t test and Mann-Whitney u test, respectively, were used, what appropriate.

* p<0.05 vs. diabetic vehicle.

Induction of diabetes by STZ led to significantly lower plasma cystatin C compared with non-diabetic controls (p<0.01), indicating that kidneys were still hyperfiltrating. This effect was prevented by treatment with riociguat, alone and in combination with telmisartan (Figure 1C). However, none of the treatments led to higher plasma cystatin C than the non-diabetic controls and therefore did not worsen kidney function.

### Plasma biomarkers

**Figure 2:**
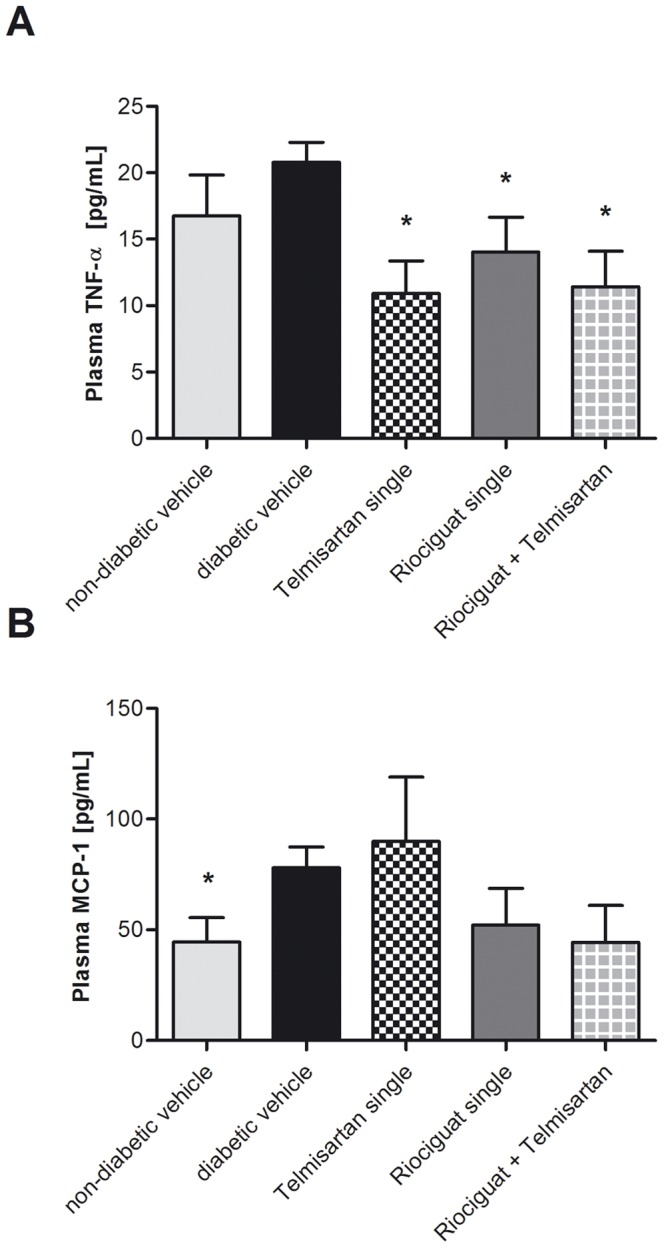
Final plasma parameters in treatment groups (riociguat, telmisartan, combination of both), diabetic and non-diabetic eNOS knockout mice. Values are given as means ± SEM. For comparisons, student's t test and Mann-Whitney u test, respectively, were used. ^*^ p<0.05 vs. diabetic vehicle. Abbreviations used: **MCP-1**, monocyte chemoattractant protein-1; **TNF-α**, tumor necrosis factor-alpha.

Treatment by solely riociguat, solely telmisartan and combined treatment significantly reduced plasma levels of TNF-α compared to diabetic control mice (Figure 2A). Whereas there was no significant difference between diabetic and non-diabetic controls for TNF-α, levels of MCP-1 were significantly decreased in non-diabetic mice compared to diabetic mice (Figure 2B). Furthermore, solely riociguat treatment as well as combined treatment with riociguat and telmisartan led to a reduced level of MCP-1 in plasma compared to diabetic mice even though missing statistical significance (p = 0.181 and p = 0.081, respectively). There was no difference in plasma MCP-1 or TNF- α when comparing mono-telmisartan and combined (riociguat and telmisartan) treatment.

### Organ weight and histology

**Figure 3:**
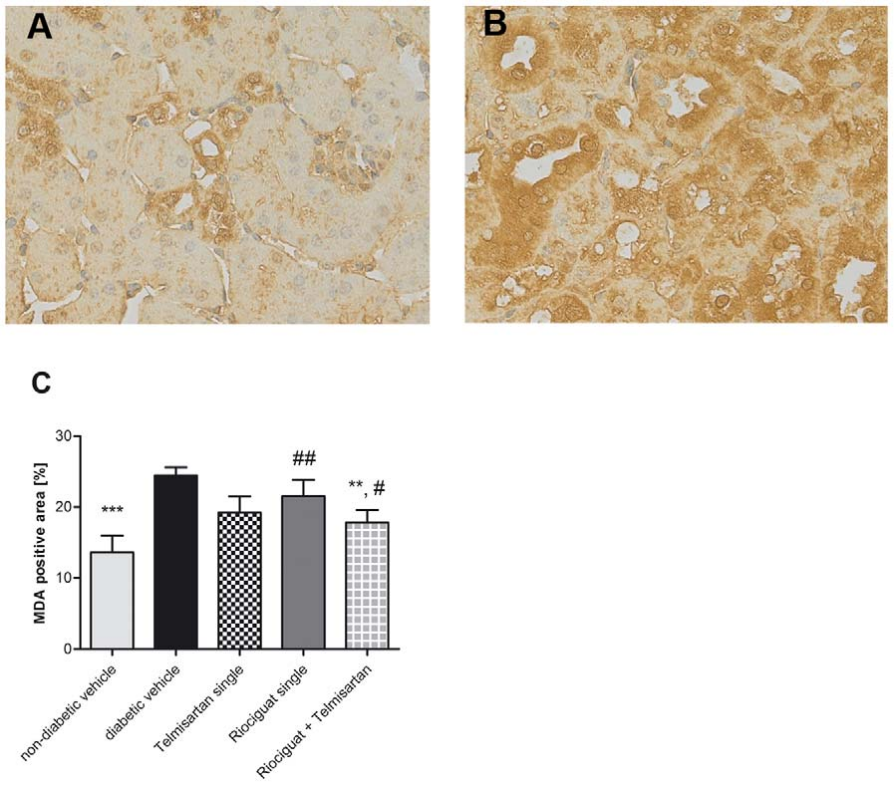
Significant reduction of MDA- positive renal tissue by combination therapy: Immunhistochemical detection of MDA in non-diabetic (A) and diabetic (B) untreated eNOS knockout mice. Percent volume of MDA-positive area in renal cortex (F). Values are given as means ± SEM. For comparisons, student's t test was used. ^**^ p<0.01; ^***^p<0.001 vs. diabetic vehicle and ^#^p<0.05; ^##^p<0.01 vs. non-diabetic vehicle. Abbreviations used: **MDA**, malondialdehyde.

Relative weights of kidney and liver were significantly higher in diabetic animals than in non-diabetic ones. However, there were no significant treatment associated differences. Perivascular fibrosis and media lumen ratio of blood vessels in the kidneys revealed no differences between the study groups (data not shown). Interstitial fibrosis was highest in untreated diabetic mice (0.8±0.1%; Table 2), followed by solely telmisartan treated animals (0.7±0.1%) and significantly decreased by riociguat (both alone and in combination with telmisartan) to a similar level of non-diabetic control mice (p = 0.014 and p = 0.049, respectively). Glomerulosclerosis appeared to be less prominent in mice treated with riociguat and/or telmisartan than in diabetic control mice. Regarding renal immunohistochemical staining for glomerular and interstitial TGF-β and CD68 expression no differences among study groups could be seen (data not shown). In contrary, staining for MDA, a marker of oxidative stress, revealed a significant increase of MDA positive tissue when comparing untreated diabetic and non-diabetic animals (p<0.001; Figure 3). All treated groups appeared to show reduced amounts of renal MDA detected by immunohistochemistry, but only the combination of riociguat and telmisartan reached significance in comparison to diabetic vehicle (p<0.01). Diabetic mice solely treated with telmisartan revealed no significant difference in MDA-detection compared with mice treated with combination of riociguat and telmisartan.

## Discussion

The aim of the present study was to investigate the effect of the novel sGC stimulator riociguat alone and in combination with the commonly used ARB telmisartan, on the progression of diabetic nephropathy in an adequate mouse model. We showed that riociguat in combination with telmisartan significantly reduced urinary albumin excretion after 11 weeks of treatment, compared with untreated diabetic mice almost reaching levels of non-diabetic control mice. Furthermore, treatment with riociguat alone and in combination with telmisartan led to a significantly reduced progression of renal interstitial fibrosis. Any treatment significantly reduced systemic inflammation as measured by plasma TNF-α levels.

Diabetes is closely associated with nephropathy and end stage renal disease [22]. However, epidemiological investigations showed that merely a percentage of diabetic patients developed nephropathy despite optimal therapy including adjustment of blood glucose, blood pressure and blockade of the renin-angiotensin-aldosterone system using ACEIs and ARBs. Consequently, further pathogenetic mechanisms must be involved, and endothelial damage is one being discussed intensively [13], [14]. Endothelial NO synthase (eNOS) activity is an important vascular modulator which is altered in diabetes, and functionally significant polymorphisms of the eNOS (NOS3) with lower production of NO [23]–[25] are associated with the development of nephropathy in patients with type 1 and type 2 diabetes [26]–[28]. Kosugi *et al.* [28] have recently shown that RAS blockade ameliorated renal injury in diabetic wild type mice but not in diabetic eNOS knock out mice, indicating that impaired NO bioavailability was responsible for resistance to RAS blockade. Accordingly, alternative stimulation of the NO/sGC/cGMP pathway offers a promising target in the treatment of diabetic nephropathy.

We show here, that riociguat in combination with telmisartan significantly reduced urinary albumin excretion and urinary albumin/creatinine ratio compared with diabetic control animals, whereas telmisartan alone led to a slight and non-significant reduction. Additionally, comparison of the combination group with telmisartan alone revealed a trend to lower urinary albumin excretion (p = 0.090). Taken together, these results indicate a potential benefit of riociguat in ARB resistant individuals.

Albuminuria is an early detection and progression marker of nephropathy in diabetic or hypertensive patients, preceding plasma cystatin C and creatinine [8], [29], [30]. Furthermore, albuminuria is associated with cardiovascular and renal morbidity and mortality [31]–[33] and is, therefore, widely used in clinical diagnostics and of huge impact. Histological analyses support our findings concerning albuminuria. Renal tubulointerstitial fibrosis was significantly reduced in animals which had received riociguat, alone and in combination, again reaching scores similar to those of non-diabetic controls. Furthermore glomerulosclerosis appeared to be less prominent in animals treated with riociguat and/or telmisartan than in vehicle-treated controls, but this finding did not show statistical significance. Although DN is commonly understood as glomerular disease, renal function correlates best with the degree of tubulointerstitial fibrosis [34] underlining the beneficial effects of riociguat treatment.

We chose male C57BL/6J mice with an eNOS (Nos3) gene knock out and administered STZ to induce diabetes mellitus. Interestingly, hyperglycemia *per se* does not necessarily cause the typical morphological and functional changes associated with DN, but in combination with the functional impairment of eNOS rodents develop albuminuria, decline of glomerular filtration rate (GFR), and renal histological pathologies such as glomerular and tubularinterstial fibrosis, similar to human DN [10]–[12], [28]. Furthermore, endothelial impairment with reduced bioavailability of NO occurs in diabetic patients as well and, moreover, its appearance is discussed as a decisive factor whether or not diabetes leads to overt nephropathy [13], [14]. Application of STZ in the present study highly increased levels of blood glucose in diabetic animals and conversely, animals which had only received citrate buffer instead of STZ were all non-diabetic (<200 mg/dL) confirming an adequate mouse model. However treatment did not have any significant influence on blood glucose.

The experiment was finished at an early stage of diabetic nephropathy as proven by plasma cystatin C levels which were lower in STZ-treated animals compared with non-diabetic controls [35]. The decline of cystatin C signaled hyperfiltration which is typical in early diabetes mellitus and significantly contributes to the progression of overt diabetic nephropathy [36]. Interestingly, riociguat, alone and in combination, abolished hyperfiltration attenuating hyperglycemic damages in kidneys.

As we have finished the experiment at an early stage, this might have resulted in less prominent differences between the study groups and, consequently, the lack of statistical significance concerning glomerulosclerosis findings. In particular, immunohistochemistry of often discussed TGFβ-pathway for the development of DN [37] only revealed slight differences between diabetic and non-diabetic animals and no benefits due to treatment could be seen. Interestingly, any treatment reduced plasma TNF alpha as a surrogate of systemic inflammation which often accompanies diabetic vasculopathy [38]. In line with this finding MCP-1 was also reduced in riociguat treated animals compared to diabetic controls by trend even though missing statistical significance. However, the assumption that MCP-1 is involved in pathological tissue remodeling could not be confirmed by immunohistochemistry.

Riociguat, alone and in combination, significantly reduced systolic blood pressure compared with vehicle treated diabetic animals, whereas the reduction achieved with telmisartan alone was slight, but not significant. This was expected since telmisartan was given in a dosage that did not intend to lower blood pressure and affect the primary endpoint albuminuria [28]. Admittedly, in clinical practice RAS blockers are also intended to lower BP and are therefore administered in dosages as high as possible. However, the increase of RAS blocker dosage does not necessarily lower SBP in diabetic eNOS mice [28]: Enalapril in dosages up to 50 mg/kg body weight showed at most only transient SBP lowering effects, whereas telmisartan, which was given in a similar dosage to ours of 2 mg/kg, only slightly reduced SBP. In the light of these results, it appears to be unlikely that we would have seen further SBP lowering effects if we had increased the dosage of telmisartan. Furthermore, neither telmisartan (2 mg/kg) nor enalapril (independently of the chosen dosage) were able to effectively reduce albuminuria and renal injuries [28]. These findings stand in line with our results and support the therapeutical insufficiency of RAS blockade in this model, independently of SBP control. . However, we cannot proof that the maximal telmisartan effect was achieved, since we did not conduct a dose response experiment

The functional loss of eNOS activity with consecutive lower bioavailability of the potent vasodilator NO leads to hypertension in mice [11], [12]. Consequently, the extrinsic stimulation of the NO/sGC/cGMP pathway by the means of riociguat effectively lowers systolic blood pressure in eNOS^−/−^ mice and has been shown previously [17]. As hypertension is a risk factor for the development of diabetic nephropathy as well, the beneficial effects of riociguat in this study may be attributed to blood pressure lowering effects. Nevertheless, recent data confirm that sGC stimulation protects against injury independently of vascular effects at a dose below that affecting blood pressure. There is evidence from animal models of hypertension indicating a protective effect against end-organ damage by stimulation of sGC independently of its hemodynamic effects. A low dose of the sGC stimulator BAY 41–2272 (close chemical analogue of riociguat) that did not affect blood pressure attenuated cardiac fibrosis in rat models of hypertension induced by infusion of angiotensin II [39] and suprarenal aortic constriction [40]. In addition, BAY 41–2272 inhibited angiotensin-converting enzyme synthesis and myofibroblast transformation in cultured cardiac fibroblasts, suggesting a mechanism by which sGC stimulation might mediate a direct antifibrotic effect in the heart [40]. Finally, a recent study in aged spontaneously hypertensive rats showed that BAY 41–2272 could completely reverse established cardiac fibrosis and reduce cardiac hypertrophy at a dose that did not produce an antihypertensive effect [41]. The sGC activators cinaciguat and ataciguat have also shown pressure-independent antiremodeling effects in the heart [41],[42]. Taken together, these results indicate that sGC agonists can exert renal and cardiac protection and that their antifibrotic effect may occur independently of their effect on vascular tone. This has important implications not only for the treatment of systemic arterial hypertension, but also for preventing its progression to cardiac dysfunction, heart failure, and renal failure.

Based on the previously reported efficacy of NO donors in preventing matrix accumulation and tissue injury [43], recent experimental studies evaluated whether an elevation in intracellular cGMP by direct stimulation of sGC would ameliorate renal disease. Administering of the sGC stimulator BAY 41-2272 to rats with an acute form of glomerulonephritis attenuated renal dysfunction. This was demonstrated by reduced proteinuria and correlated with decreased TGFβ production, matrix deposition and macrophage infiltration [44]. A subsequent study demonstrated that BAY 41-2272 elevated cGMP levels in mesangial cells, thereby reducing their proliferation and matrix production [45]. Interestingly, the disease process itself upregulates sGC protein expression with a concomitant increase in cGMP levels, indicating that this effect could represent an endogenous protective mechanism, which adds further weight to the validity of utilizing pharmacological sGC stimulators to prevent renal disease. Subsequent investigations in a chronic model of glomerulonephritis confirmed that BAY 41-2272 protects the kidney from progressive sclerosis and matrix deposition by limiting TGFβ expression [46], [47]. The protective effect achieved by elevating cGMP via direct sGC stimulation with BAY 41-2272 was far superior to that produced by preventing degradation of cGMP using the PDE inhibitor pentoxifylline.

Oral BAY 41-2272 and BAY 41–8543 also produced dose-dependent vasodilation and markedly improved survival in rat models of hypertension without causing tolerance [48], [49]. Furthermore, studies in low-NO rat models of hypertension demonstrated that BAY 41-8543 had a renal protective effect. BAY 41-2272 attenuated cardiac fibrosis and hypertrophy [50], and riociguat provided significant protection against cardiac and renal damage, reducing glomerulosclerosis, cardiac and renal interstitial fibrosis, and normalized left ventricular weight. The sGC stimulator riociguat also normalized blood pressure and demonstrated renal and cardiac protective effects in a rat model of chronic renal failure [17]. In a model of pressure and volume overload (Dahl salt-sensitive rats on a high salt diet) treated with riociguat markedly attenuated systemic hypertension, improved systolic heart function and increased survival from [18]. Histological examination of the heart and kidneys revealed that riociguat significantly ameliorated fibrotic tissue remodeling and degeneration in the myocardium and the renal cortex. Moreover, sGC stimulation by BAY 41-8543 increases cGMP production and subsequently enhances renal recovery after unilateral ureteral obstruction relief and may serve as a novel treatment approach to restore or preserve renal structure and function in cases of obstructive kidney disease.

Recent data confirm that sGC stimulation may protect against organ injury independently of its effects on vascular tone: at a dose below that affecting blood pressure, BAY 41-2272 still attenuated cardiac fibrosis in rodent models of hypertension induced by infusion of angiotensin II [39] and suprarenal aortic constriction [40]. These pleiotropic effects are mediated via decreased collagen accumulation, reduced myofibroblast numbers, and inhibition of transforming growth factor beta 1 and type 1 collagen gene expression, which result from increases in the intracellular cGMP concentration. This strongly suggests that these new compounds are capable of restoring physiological signaling in extravascular cells, including the endothelium-mediated regulation of myocardial and renal function.

Our study clearly demonstrated that only the combination of riociguat and telmisartan is able to significantly reduce renal malonaldehyde immunreactivity in diabetic eNOS knock out mice. Reactive oxygen species degrade polyunsaturated lipids, forming malondialdehyd, a reactive aldehyde, that is a reactive electrophile species that causes toxic stress in cells and forms covalent protein adducts referred to as advanced lipoxidation end-products (ALE) [51]. Malondialdehyde is thus a biomarker of the tissue concentration of reactive oxygen species and its tissue damage. Oxidative stress is thought to play a major role in the pathogenesis of diabetic nephropathy [52]–[54]. Our data suggest that the beneficial effect of the combination treatment of diabetic eNOS knock out mice using riociguat and telmisartan on albumin excretion might be at least partially attributed to a decrease in oxidative stress.

Further studies are needed to clarify the molecular mechanisms of beneficial pleiotropic effects of riociguat via the NO/sGC/cGMP pathway. Especially the molecular mechanisms leading to a decrease in oxidative stress need to be investigated, since this seems to be key in the understanding of the effectiveness of the combination therapy. A study limitation is the fact that RAS blockade might have been insuffient. However, other independent working groups found similar insufficient effects at even higher dosages [28]. Furthermore, we cannot prove that the therapeutic benefits presented in this study were independent of blood pressure which is a well-described predictor of DN. On the other hand, we provide an important step towards the clinical evaluation of the cardio-renal protective properties of sGC stimulators. Our study is in particular important with aspects of translation to clinical studies. Nowadays, new compounds for the treatment of diabetic nephropathy need to show further benefit on top of guideline based therapy with ARBs in type 2 diabetic patients. With respect to interstitial fibrosis, which is an important predictor of chronic renal failure [55]–[57], our paper presents a compelling case for riociguat, which might be of clinical importance in patients who show contraindications for RAS blockade, such as renal artery stenosis or kidney transplantation. The strength of our study is that we were close to this clinical situation in our experimental design by choosing an animal model that is close to the human situations as well as by showing that our compound enhances the effect of an ARB. Thus our data should clearly stimulate further clinical work.

### Conclusion

Stimulation of soluble guanylate cyclase by riociguat significantly reduced urinary albumin excretion, a very early biomarker of diabetic nephropathy, in diabetic eNOS knockout mice on top of ARB treatment. As patients with diabetic nephropathy refractory to treatment with ARBs have the worst prognosis among all patients with diabetic nephropathy, our findings may offer a new therapeutic approach for those patients.
